# Strukturelle Versorgungssituation der operativen Therapie bei männlicher Belastungsinkontinenz in Deutschland

**DOI:** 10.1007/s00120-024-02360-0

**Published:** 2024-05-29

**Authors:** Viktoria Menzel, Ricarda M. Bauer, Markus Grabbert, Juliane Putz, Nicole Eisenmenger, Luka Flegar, Angelika Borkowetz, Johannes Huber, Christian Thomas, Martin Baunacke

**Affiliations:** 1 Klinik und Poliklinik für Urologie, Universitätsklinikum Carl Gustav Carus an der Technischen Universität Dresden, Fetscherstr. 74, 01307 Dresden, Deutschland; 2 Klinik und Poliklinik für Urologie, LMU Klinikum, Campus Großhadern, München, Deutschland; 3 Klinik für Urologie, Universitätsklinikum Freiburg, Freiburg, Deutschland; 4 Reimbursement Institut, Hürth, Deutschland; 5 Klinik für Urologie, Philipps-University Marburg, Marburg, Deutschland

**Keywords:** Inkontinenzoperationen, Artifizielle Sphinkter, Schlinge, Versorgung, Radikale Prostatektomie, Incontinence surgery, Artificial sphincter, Male sling, Health care, Radical prostatectomy

## Abstract

**Hintergrund:**

Die Belastungsinkontinenz des Mannes ist überwiegend iatrogen bedingt. Aktuelle Studien zeigen nicht nur in Deutschland ein Versorgungsdefizit in der operativen Therapie. Ziel ist es, die strukturelle Versorgung der operativen Therapie der männlichen Belastungsinkontinenz in Deutschland detaillierter zu untersuchen.

**Material und Methoden:**

Die Auswertung der strukturellen Versorgung durch Belastungsinkontinenzoperationen des Mannes in Deutschland erfolgt anhand von OPS-Codes der Qualitätsberichte der Krankenhäuser von 2011 bis 2019.

**Ergebnisse:**

Von 2012 bis 2019 zeigt sich ein Rückgang der Inkontinenzoperationen beim Mann von 2191 auf 1445. Die Zahl der Kliniken, die Inkontinenzoperationen durchführen, fiel von 275 auf 244. In der multivariaten Analyse ist eine hohe Zahl (*n* ≥ 50) an radikalen Prostatektomien/Jahr (RPE/Jahr) ein unabhängiger Prädiktor für eine High-volume-Klinik (≥ 10 Eingriffe/Jahr: Odds Ratio [OR] 6,4 [2,3–17,6]; *p* < 0,001). Ein deutlicher Rückgang ist bei Schlingenoperationen (von *n* = 1091 auf 410; *p* < 0,001) zu verzeichnen. Hier sank besonders die Fallzahl in Kliniken, die eine hohe Zahl an Schlingen implantierten (≥ 10 Schlingen/Jahr; −69 %; −62,4 ± 15,5 Operationen/Jahr; *p* = 0,007). Diese haben sich auch in ihrer Anzahl verringert (von *n* = 34 auf 10; *p* < 0,001). Dies betraf insbesondere Klinken, die auch eine geringe Zahl an RPE/Jahr aufwiesen (Zahl der Kliniken von 9 auf 0 gefallen [−100 %]).

**Schlussfolgerung:**

Die Versorgungssituation der operativen Therapie der männlichen Belastungsinkontinenz in Deutschland zeigt einen deutlichen Rückgang der Schlingenimplantation, insbesondere in kleinen Kliniken. Auf der einen Seite reflektiert dies die zunehmend differenzierte Indikationsstellung der Schlingenimplantation. Auf der anderen Seite ergibt sich der Verdacht auf eine entstandene Versorgungslücke, da eine Kompensation durch andere operative Verfahren nicht ersichtlich ist.

## Abstract (en)

**Background:**

Stress urinary incontinence in men is predominantly iatrogenic after radical prostatectomy or transurethral interventions. Current studies show that there is a deficit in the availability of surgical therapy not only in Germany. The aim of this study is to investigate in more detail the structural health care situation of surgical treatment of male stress incontinence in Germany.

**Materials and methods:**

The evaluation of the surgical therapy of male stress incontinence in Germany is based on the OPS (Operationen- und Prozedurenschlüssel—German procedural classification) codes from hospital quality reports from 2011–2019.

**Results:**

From 2012–2019, the number of male incontinence surgeries declined from 2191 to 1445. The number of departments performing incontinence surgeries decreased from 275 to 244. In the multivariate analysis, a high number (≥ 50) of radical prostatectomies/year (RPE/year) is an independent predictor of a high-volume centre (≥ 10 procedures/year; odds ratio [OR] 6.4 [2.3–17.6]; *p* < 0.001). The most significant decrease was in sling surgery (from 1091 to 410; *p* < 0.001). Here, the number of cases decreased especially in departments that implanted a high number of slings (≥ 10 slings/year; −69%; −62.4 ± 15.5 surgeries/year; *p* = 0.007). In addition, the number of departments implanting slings decreased over the investigated time period (from 34 to 10; *p* < 0.001). This particularly affected departments that also had a low number of RPE/year (from 9 to 0; −100%).

**Conclusion:**

The situation of surgical treatment of male stress urinary incontinence in Germany shows a clear decline in sling implantation, especially in small departments. On the one hand, this reflects the increasingly differentiated indications for sling implantation. On the other hand, it raises the suspicion that a gap in care has developed, as the decline was not compensated for by other surgical therapies.

## Einleitung

Die Harninkontinenz stellt eine deutliche Belastung der Lebensqualität dar [1]. Die Belastungsinkontinenz des Mannes ist dabei überwiegend iatrogen bedingt, wobei die häufigste Ursache die radikale Prostatektomie (RPE) mit Inkontinenzraten zwischen 4–31 % [2–4] ist. Es sollte dem urologischen Fachbereich ein besonderes Anliegen sein, diese Folgeschädigung von urologischen Eingriffen adäquat zu behandeln. Nach erfolgloser konservativer Therapie steht hier eine Reihe von operativen Verfahren zur Verfügung [5]. Während Schlingen und Schlingensysteme bei leicht- bis mittelgradiger Belastungsinkontinenz eingesetzt werden können, werden artifizielle Sphinkter bei mittel- bis schwergradiger Belastungsinkontinenz implantiert [6]. Auch wenn die verschiedenen Implantate ein relevantes Komplikationsprofil aufweisen, zeigen sie gute Ergebnisse in der Reduktion der Inkontinenz und der Verbesserung der Lebensqualität [7, 8].

Eine Auswertung inkontinenter Männer nach RPE im Rahmen der HAROW-Studie (*H*ormontherapie, *A*ctive *S*urveillance, *R*adiotherapie, *O*peration, *W*atchful Waiting) zeigt, dass nur 46 % der Inkontinenten mit einem hohen Leidensdruck operativ versorgt werden [9]. Eine aktuelle britische Studie zeigt ebenfalls ein Defizit auf. Hier erhielten nur 20 % der inkontinenten Männer nach RPE mit hohen Leidensdruck eine operative Therapie [10]. Weiterhin zeigen eine Reihe von internationalen Datenbankauswertungen eine niedrige Rate an Inkontinenzoperationen nach RPE (2,5–3,9 %), die deutlich niedriger ist als die zu erwartende Inkontinenzrate [10–14]. Aufgrund dieser Daten führte unsere Arbeitsgruppe eine Analyse der Versorgungsrealität von Inkontinenzoperationen beim Mann von 2006 bis 2020 in Deutschland durch. Hier zeigt sich ab 2012 ein zunehmender Rückgang der Inkontinenzoperationen beim Mann. Dieser ist im Wesentlichen durch den deutlichen Rückgang von Schlingenimplantationen bedingt [13]. Weitere Untersuchungen sind notwendig, um die Gründe für die Unterversorgung und den Rückgang von Inkontinenzoperationen herauszufinden. Hierfür sind patienten- und arztzentrierte Erhebungen notwendig. In der folgenden Arbeit soll zunächst die strukturelle Versorgung der operativen Therapie der männlichen Belastungsinkontinenz in Deutschland detaillierter untersucht und die Bedeutung des Rückgangs der Schlingenimplantationen eingeordnet werden.

## Material und Methoden

Die Auswertung der strukturellen Versorgung von Belastungsinkontinenzoperationen des Mannes in Deutschland erfolgt anhand der Qualitätsberichte der Krankenhäuser. Die Qualitätsberichte sind seit 2005 gesetzlich verpflichtend. Zur Extraktion dieser Daten wurde das Analysetool reimbursement.INFO (RI Innovation GmbH, Hürth) genutzt. Die Operationen- und Prozedurenschlüssel (OPS-Kodes) wurden für urologische Kliniken (Fachabteilungsschlüssel 2200) als Einfachkodierungen extrahiert. Bei Qualitätsberichten werden niedrige Fallzahlen von 1–3 Fällen/Jahr anonymisiert und als 1 angegeben. Somit ist eine absolute exakte Angabe von Fallzahlen nicht möglich. Weiterhin wird in Qualitätsberichtsdaten nicht das Geschlecht angegeben, weswegen für die folgenden Einschätzungen die Daten des statistischen Bundesamtes (DESTATIS) genutzt wurden. Hier wurde der Frauenanteil bei den OPS-Kodes im Jahr 2019 ermittelt, um zu beurteilen, wie valide die Nutzung der OPS-Kodes der Qualitätsberichte zur Beurteilung von Eingriffen beim Mann sind. Für die Implantation oder Wechsel eines artifiziellen Sphinkters wurden folgende Kodes genutzt: 5‑597.0, 5‑597.30, 5‑597.31, 5‑597.32. Hier betrug der Frauenanteil 1,5 % im Jahr 2019. Für die Implantation von Schlingen bzw. Schlingensystemen gibt es mehrere Kodierungen, deren Anwendung ungenau definiert ist. Folgende Kodes wurden für diese Studie analysiert: 5‑598.0 und 5‑598.x (Frauenanteil 0,9 %), sowie 5‑596.75 (Frauenanteil 0,8 %). Der Kode 5-596.75 existiert seit 2014. Davor wurde der unspezifische Kode 5-596.70 genutzt. Dieser hat einen Frauenanteil von 55,8 % im Jahr 2012. Entsprechend der Qualitätsberichte wurde der Kode in 63,9 % in gynäkologischen Fachabteilungen genutzt, sodass auch hier der Frauenanteil in der Analyse von urologischen Fachabteilungen nicht relevant ist. Der Kode 5-594 wurde nicht in die Analyse miteingeschlossen, da hier wiederum der Anteil an Männern mit 2,0 % verschwindend gering ist.

Der Kode 5-604 wurde genutzt zur Ermittlung der Zahl an radikalen Prostatektomien in den Kliniken.

Es erfolgte eine Analyse der Inkontinenzoperationen insgesamt sowie eine getrennte Analyse von Sphinkter- und Schlingenimplantationen und eine Unterteilung der Kliniken in „high volume“ und „low volume“. High-volume-Kliniken führen ≥ 10 Eingriffe/Jahr durch. Low-volume-Kliniken führen 1–9 Eingriffe/Jahr durch.

Die Analyse erfolgte zwischen 2012 und 2019. Während dieser Zeit nahmen die Inkontinenzoperationen in Deutschland stetig ab. Das Jahr 2020 wurde aufgrund des Einflusses der Coronapandemie nicht berücksichtigt [13].

Die Kartendarstellung erfolgte mit der Software easymap© office (Lutum + Tappert DV-Beratung GmbH, Bonn). Die statistische Auswertung erfolgte mit t‑Test, χ^2^-Test, uni- und multivariater binärer logistischer Regression, sowie mit linearer Regressionsanalyse. Die Berechnungen erfolgten mit „IBM SPSS Statistics 28“ (IBM, Armonk, NY, USA).

## Ergebnisse

### Strukturelle Versorgung in Deutschland

**Abb. 1:**
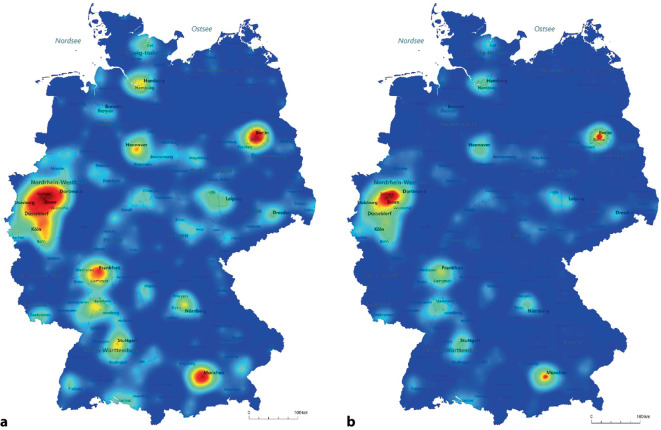
Heatmap zur Dichte an männlichen Inkontinenzoperationen in Deutschland 2012 (**a**) und 2019 (**b**)

Im Jahr 2012 wurden 2191 Inkontinenzoperationen bei Männern in 275 Kliniken durchgeführt. Der Anteil der High-volume-Kliniken betrug 23,6 % (*n* = 65). Diese führten 67,5 % (*n* = 1480) aller Eingriffe durch. Im Jahr 2019 wurden 1445 Inkontinenzoperationen bei Männern in 244 urologischen Kliniken durchgeführt. Von diesen Kliniken waren 15 % (*n* = 37) High-volume-Kliniken (≥ 10 Inkontinenzoperationen/Jahr). Diese führten 62,7 % (*n* = 907) aller Eingriffe durch (Tab. 1). Die Verteilung der Zahl an Inkontinenzoperationen in Deutschland geht einher mit der Bevölkerungsdichte. Es zeigt sich eine hohe Dichte an Operationen im Westen, Südwesten sowie in den Ballungsräumen München, Nürnberg, Berlin, Hamburg und Hannover. Eine geringe Dichte weist Mitteldeutschland und insbesondere der Nordosten auf (Abb. 1).

**Tab. 1:** Kollektiv urologischer Fachabteilungen in Deutschland im Jahr 2019 verteilt nach ihrer Fallzahl an männlichen Inkontinenzoperationen („low volume“ vs. „high volume“)

		Gesamt (*n* = 244)	Low-volume-Klinik (1–9) (*n* = 207)	High-volume-Klinik (≥ 10) (*n* = 37)	*p*-Wert
Deutschland	Ost	60 (25 %)	54 (26 %)	6 (16 %)	0,2
	West	184 (75 %)	153 (74 %)	31 (84 %)	
Universitätsklinik	Nein	212 (87 %)	187 (90 %)	25 (68 %)	**<** **0,001**
	Ja	32 (13 %)	20 (10 %)	12 (32 %)	
Zertifiziertes Beckenbodenzentrum	Nein	189 (77 %)	162 (78 %)	27 (73 %)	0,5
	Ja	55 (23 %)	45 (22 %)	10 (27 %)	
Stadtgröße	< 50.000	92 (38 %)	85 (41 %)	7 (19 %)	**0,02**
	50.000–499.999	108 (44 %)	89 (43 %)	19 (51 %)	
	≥ 500.000	44 (18 %)	33 (16 %)	11 (30 %)	
RPE-Fallzahl 2019	< 50	123 (50 %)	118 (57 %)	5 (14 %)	**<** **0,001**
	≥ 50	121 (50 %)	89 (43 %)	32 (86 %)	
RPE-Fallzahl absolut 2019		64,4 ± 126,9 29 (0–1.393)	50,1 ± 95,6 26 (0–1.325)	220,8 ± 259,8 119 (0–1.393)	**<** **0,001**

*fettgedruckt*: statistisch signifikant

*RPE* radikale Prostatektomie

High-volume-Kliniken sind im Jahr 2019 signifikant häufiger Universitätskliniken (32 % vs. 10 %; *p* < 0,001), weisen größtenteils ≥ 50 RPE/Jahr auf (86 % vs. 43 %; *p* < 0,001) und liegen vermehrt in Großstädten (30 % vs. 16 %; *p* = 0,02; Tab. 1). In der multivariaten Analyse zeigt sich, dass eine hohe Fallzahl an RPE (≥ 50 Eingriffe/Jahr) ein unabhängiger Prädiktor für eine High-volume-Klinik ist (Tab. 2).

**Tab. 2:** Uni- und multivariate Analyse der Prädiktoren für High-volume-Kliniken von Inkontinenzoperationen beim Mann im Jahr 2019

	Univariate Analyse		Multivariate Analyse	
	OR (95 %-KI)	*p*-Wert	OR (95 %-KI)	*p*-Wert
Stadtgröße (≥ 500.000)	2 (1,2–3,2)	**0,006**	1,3 (0,7–2,4)	0,3
RPE-Fallzahl (≥ 50)	8,5 (3,1–22,7)	**<** **0,001**	6,4 (2,3–17,6)	**<** **0,001**
Universitätsklinik	4,5 (2,0–10,3)	**<** **0,001**	2,4 (1,0–5,8)	0,06
Westdeutschland	1,8 (0,7–4,6)	0,2	–	–
Zertifiziertes Beckenbodenzentrum	1,3 (0,6–3,0)	0,5	–	–

*RPE* radikale Prostatektomien, *KI* Konfidenzintervall, *OR* Odds Ratio

In 93 Kliniken ist die Zahl an Inkontinenzoperationen um ≥ 5 Eingriffe/Jahr von 2012 auf 2019 gesunken. In 23 Kliniken hat sich die Zahl der Eingriffe um ≥ 5/Jahr von 2012 auf 2019 erhöht. Von 2012 bis 2019 hat sich der Anteil an High-volume-Kliniken verringert (24 % auf 15 %, *p* = 0,02) sowie deren anteilige Fallzahl (68 % auf 63 %, *p* = 0,003). Die durchschnittliche Zahl an Eingriffen/Klinik hat sich an High-volume-Kliniken erhöht (von 22,8 auf 24,5 Operationen/Jahr und Klinik) und an Low-volume-Kliniken verringert (von 3,4 auf 2,6 Operationen/Jahr und Klinik; Tab. 3).

**Tab. 3:** Entwicklung von Low- und High-volume-Kliniken von Inkontinenzoperationen von 2012 bis 2019

		2012	2019	*p*-Wert
Klinikzahl	„Low volume“	210 (76 %)	207 (85 %)	**0,02**
	„High volume“	65 (24 %)	37 (15 %)	
Fallzahl	„Low volume“	711 (32 %)	538 (37 %)	**0,003**
	„High volume“	1.480 (68 %)	907 (63 %)	
Anzahl Fallzahl/Klinik	„Low volume“	3,4	2,6	n. s.
	„High volume“	22,8	24,5	

### Getrennte Entwicklung der Operationsverfahren in Deutschland

**Abb. 2:**
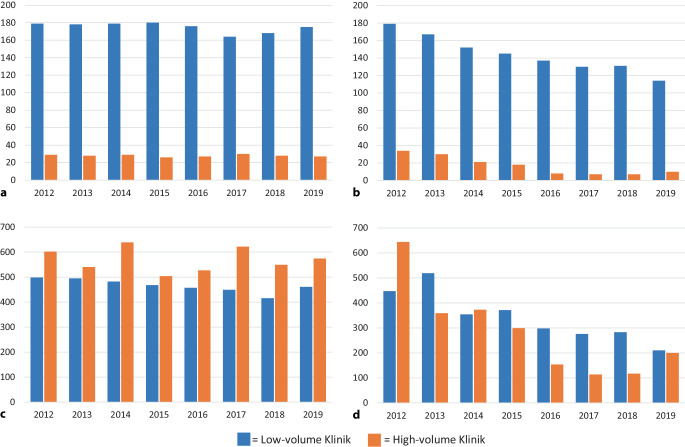
Entwicklung der Klinik- und Fallzahl von 2012 bis 2019 in Low-volume- (1–9 Eingriffe/Jahr) und High-volume- (≥ 10 Eingriffe/Jahr) Kliniken in Deutschland bezogen auf Sphinkter- und Schlingenimplantationen: **a** Sphinkter: Zahl der Kliniken pro Jahr; **b** Bänder: Zahl der Kliniken pro Jahr; **c** Sphinkter: Fallzahl pro Jahr; **d** Bänder Fallzahl pro Jahr

Von 2012 bis 2019 zeigt sich bei Sphinkterimplantationen eine stabile Fallzahl (2012: 1100; 2019: 1035). Die Zahl an implantierenden Low-volume- und High-volume-Kliniken ist ebenfalls stabil geblieben (*p* = 0,09 & *p* = 0,6). Auch die Fallzahl zeigt bei Low-volumen-Kliniken (−9,1 ± 2,5 Operationen/Jahr, *p* = 0,01) und bei High-volume-Kliniken (−2,1 ± 7,9; *p* = 0,8) eine geringe bzw. keine Veränderung (Abb. 2).

Von 2012 bis 2019 zeigt sich bei Schlingenimplantationen ein Rückgang der Fallzahlen (von 1091 auf 410; *p* < 0,001). Außerdem sinkt die Zahl der Low-volume-Kliniken (−36 %; −8,4 ± 0,7 Kliniken/Jahr; *p* < 0,001) und der High-volume-Kliniken (−71 %; −4,0 ± 0,7 Kliniken/Jahr; *p* = 0,002). Die Fallzahl von Schlingenimplantationen ist in High-volume-Kliniken (−69 %; −62,4 ± 15,5 Operationen/Jahr; *p* = 0,007) stärker zurückgegangen als in Low-volume-Kliniken (−53 %; −37 ± 6,8 Operationen/Jahr; *p* = 0,001; Abb. 2).

### Entwicklung von High-volume-Schlingenkliniken

**Abb. 3:**
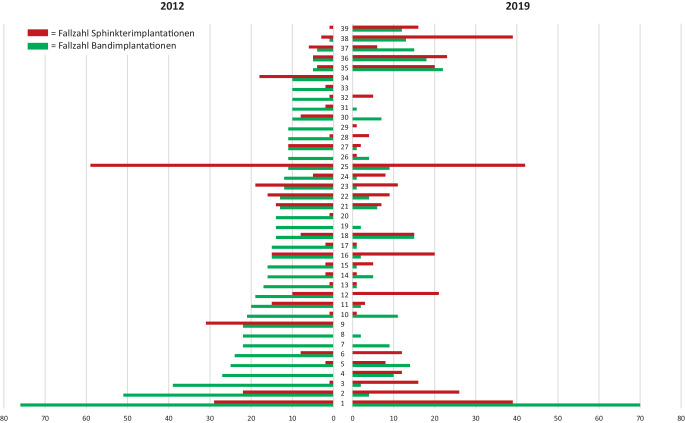
Vergleich der Fallzahlen von Schlingen- und Sphinkterimplantationen von 39 Kliniken mit High-volume-Schlingenimplantationen (≥ 10 Eingriffe/Jahr) im Jahr 2012 (1–34 Eingriffe/Jahr) bzw. 2019 (35–39 Eingriffe/Jahr)

Im Jahr 2012 implantierten 34 urologische Kliniken ≥ 10 Schlingen. Von diesen Kliniken waren im Jahr 2019 15 % (*n* = 5) weiterhin High-volume-Kliniken, 59 % (*n* = 20) haben sich zu Low-volume-Kliniken (Ø 3,8 Schlingen) entwickelt und 26 % (*n* = 9) haben keine Schlingen mehr implantiert. Von den früheren High-volume-Kliniken, die 2019 weniger oder keine Schlingen mehr implantierten (*n* = 29), haben 14 % (4/29) gar keine Inkontinenzoperationen beim Mann mehr durchgeführt, bei 34 % (10/29) ist die Zahl der Sphinkterimplantationen gleichzeitig gesunken (Ø −7,2 Sphinkterimplantationen), bei 14 % (4/29) ist die Zahl der Sphinkterimplantationen gleichgeblieben bzw. wurde bereits 2012 nicht durchgeführt. Bei 38 % (11/29) ist die Zahl der Sphinkterimplantationen gestiegen (Ø + 4,9 Sphinkterimplantationen). Im Jahr 2019 sind zu den bestehenden 5 High-volume-Kliniken 5 weitere High-volume-Kliniken hinzugekommen. Diese 5 neuen Kliniken haben die Zahl der Schlingenimplantationen/Klinik um Ø + 13 gesteigert. 4 dieser Kliniken haben auch die Sphinkterimplantationen/Klinik um Ø + 21 gesteigert (Abb. 3).

Kliniken mit einer niedrigen Fallzahl an RPE/Jahr weisen einen höheren Rückgang ihrer durchschnittlichen Fallzahl an Schlingenoperationen von 2012 bis 2019 auf als Kliniken mit hoher RPE-Fallzahl (−79 % vs. −51 % [1,4 ± 3,4–0,3 ± 0,8] vs. [4,3 ± 9,1–2,1 ± 6,6]). Kliniken mit einer niedrigen Fallzahl an RPE/Jahr weisen einen höheren Rückgang an ihrem Anteil an High-volume-Schlingenkliniken auf als Kliniken mit hoher RPE-Fallzahl (−100 % vs. −60 % [9–0 vs. 25–10]; Tab. 4).

**Tab. 4:** Vergleich der Entwicklung der Schlingenoperationen zwischen Kliniken mit niedriger und hoher RPE-Fallzahl (radikale Prostatektomie)

		Kliniken mit RPE < 50/Jahr	Kliniken mit RPE ≥ 50/Jahr
Entwicklung Fallzahl Schlingen 2012–2019		−79 % (1,4 ± 3,4 vs. 0,3 ± 0,8)	−51 % (4,3 ± 9,1 vs. 2,1 ± 6,6)
Entwicklung von Kliniken zum Jahr 2019 entsprechend ihrer Schlingenfallzahl 2012	„Low volume“ 2012	+6 % (272 vs. 288)	+6 % (137 vs. 145)
	„High volume“ 2012	−100 % (9 vs. 0)	−60 % (25 vs. 10)

*RPE* radikale Prostatektomie

## Diskussion

In der Analyse der strukturellen Entwicklung von Inkontinenzoperationen beim Mann in Deutschland zeigt sich im Jahr 2019 eine Zentralisierung mit 37 High-volume-Kliniken, die 62,7 % aller Eingriffe bundesweit durchführen. In der Beurteilung der Entwicklung der Inkontinenzoperationen beim Mann von 2012 bis 2019 zeigt sich im Gegensatz zu Sphinkterimplantationen ein deutlicher Rückgang bei Schlingenimplantationen, insbesondere in High-volume-Schlingenkliniken bzw. in Kliniken mit niedriger Zahl an RPE/Jahr.

Kliniken mit einer hohen Fallzahl an Inkontinenzoperationen pro Jahr sind signifikant häufig Kliniken mit einer hohen Fallzahl an RPE/Jahr (220,8 ± 259,8 vs. 50,1 ± 95,6; *p* < 0,001) assoziiert. In der multivariaten Analyse ist eine Fallzahl von ≥ 50 RPE/Jahr ein unabhängiger Prädiktor (OR 6,4 [2,3–17,6]; *p* < 0,001) für eine Klinik mit hoher Fallzahl an Inkontinenzoperationen (≥ 10 Operationen/Jahr). Dies kann zum einen dadurch bedingt sein, dass eine Klinik mit hoher RPE-Fallzahl auch mit mehr inkontinenten Männern in Kontakt kommt. Zum anderen spiegelt die Zahl an RPE/Jahr mutmaßlich auch das Patientenaufkommen der einzelnen Kliniken wider [15].

In der Entwicklung von 2012 bis 2019 zeigt sich eine deutliche Abnahme an Inkontinenzoperationen beim Mann. Diese Entwicklung hat sich aber anteilig ungleichmäßig auf die Zahl an High- und Low-volume-Kliniken und deren Fallzahl verteilt. 2019 werden in anteilig weniger High-volume-Kliniken durchschnittlich mehr Fälle (24,5 vs. 22,8 Operationen/Jahr) und in anteilig mehr Low-volume-Kliniken durchschnittlich weniger Fälle (2,6 vs. 3,4 Operationen/Jahr) versorgt (Tab. 3). In Hinblick auf die relevante Komplikations- und Revisionsrate insbesondere bei artifiziellen Sphinkteren ist eine Zentrumsbildung mit entsprechend hoher operativer Erfahrung zu befürworten [8, 16]. Dies scheint sich aber in der Entwicklung von 2012 bis 2019 nicht eindeutig widerzuspiegeln.

Betrachtet man die Entwicklung der beiden operativen Eingriffe (artifizieller Sphinkter vs. Schlingen/Schlingensystem) getrennt, zeigt sich hier ein differenzierteres Bild des Rückgangs der Inkontinenzoperationen von 2012 bis 2019. Es zeigt sich eine stabile Zahl an implantierenden Kliniken und deren Fallzahl bei artifiziellen Sphinkteren von 2012 bis 2019, während der relevante Rückgang bei Schlingen/Schlingensystemen zu verzeichnen ist (Abb. 2). Die Gründe für diese Entwicklung sind letztlich unklar. Von 2006 bis 2011 nahmen Schlingenoperationen bei Männern in Deutschland zu [13]. In den USA zeigte sich eine ähnliche Entwicklung von 2003 bis 2013 [17]. Eine britische Studie wies zunächst einen Anstieg bis 2014 nach, gefolgt von einen kontinuierlichen Rückgang von Schlingenoperationen beim Mann in den Folgejahren [18]. Möglicherweise lässt sich diese Entwicklung auf den initialen „sling hype“ zurückführen. Mit der Einführung von Schlingen für die männliche Belastungsinkontinenz stand ein Operationsverfahren zur Verfügung, dass sowohl für den Operateur als auch den Patienten weniger komplex ist als die Implantation eines artifiziellen Sphinkters und als minimal-invasive Alternative bewertet wurde [19, 20]. Mit zunehmender Studienlage zeigten sich aber auch mögliche Schwächen der Schlingen, im Sinne einer begrenzten Wirksamkeit bei schwerer Harninkontinenz sowie die Tatsachen, dass eine differenziertere Indikationsstellung und erweiterte Diagnostik vor Schlingenimplantation notwendig ist [6, 21]. In der strukturellen Analyse dieser Arbeit lässt sich nachvollziehen, dass der Fallzahleinbruch bei Schlingenoperationen insbesondere kleinere Kliniken betroffen hat (< 50 RPE/Jahr). Hier ist die Fallzahl deutlich zurückgegangen (−79 % vs. −51 %) und es gibt keine kleinen Kliniken mehr, die bzgl. Schlingenimplantationen High-volume-Kliniken sind (−100 % vs. −60 %; Tab. 4). Hier ist zu vermuten, dass insbesondere kleine Kliniken den gewissen „sling hype“ initial mitgenommen und auch wieder schneller verlassen haben.

Ein weiterer zu diskutierender Grund ist der Einfluss des Verbotes der FDA (Food and Drug Administration) in den USA von vaginalen Netzen [22]. Studien zeigen hier, dass das eigentliche Verbot von Netzen in der Prolapschirurgie in den Medien oftmals nicht differenziert dargestellt wird und oft auch Schlingen zur Behandlung der Harninkontinenz umfasst [23–25]. In Hinblick auf fachliche Diskussionen ist davon auszugehen, dass dieses Thema bei der männlichen Belastungsinkontinenz eher keine Rolle spielt.

Zusammengefasst stellt sich die Frage, wie der Rückgang der Schlingenimplantation zu erklären ist bzw. welche Therapie inkontinente Männer heute erfahren, die 2012 noch für eine Schlingenimplantation ausgewählt wurden. Wie in der Vorarbeit dargestellt, ist ein Rückgang an Inkontinenzoperationen eher nicht durch weniger Indikationen zu erklären, da die Zahl an RPE seit 2014 wieder steigt [13]. Es haben sich bzgl. der operativen Technik in den letzten 10 Jahren keine erhebliche Neuerung ergeben, die eine signifikante Verbesserung der Inkontinenzraten zur Folge hätte. Zum anderen nimmt der Anteil an älteren Patienten, die sich einer RPE unterziehen, zu. Insbesondere ältere Patienten weisen ein höheres Inkontinenzrisiko auf. Dies spiegelt sich auch in einem zunehmenden Anteil älterer Patienten zur Inkontinenztherapie wider [13]. Hier ergibt sich somit der Verdacht auf ein Versorgungsdefizit. Sowohl das Follow-up der HAROW-Studie als auch einer britischen Studie untermauern diesen Verdacht, indem sie zeigen, dass es einen relevanten Anteil inkontinenter Männer mit hohen Leidensdruck gibt, die aber keine Therapie erhalten haben [9, 10]. Es gibt einige Untersuchungen, die unterstreichen, dass hier ein Kommunikationsproblem zwischen Arzt und Patient bestehen könnte. Zum einen wird ärztlich der Leidensdruck der Betroffenen als weniger problematisch eingeschätzt, als es der Betroffene selbst sieht und zum anderen sprechen es die Betroffenen im Arztkontakt auch weniger als notwendig an [26–28]. In Hinblick auf die Zeit des „sling hypes“ 2011/2012 kann vermutet werden, dass betroffene Patienten häufiger auf eine operative Therapie angesprochen wurden, da ärztliche Erwartungen darauf abzielten, diesen Betroffenen mit einer Schlingenoperation unkompliziert und einfacher helfen zu können als mit der Implantation eines artifiziellen Sphinkters.

Als Gegenbeispiel zur allgemeinen Entwicklung des Rückgangs der Schlingen in Deutschland kann die LMU München betrachtet werden (Abb. 3, Nr. 1) mit einer konstant hohen Fallzahl an Schlingenoperationen. Diese hohe Fallzahl ermöglicht die entsprechende Expertise aufzubauen, die z. B. bei der komplexen Indikationsstellung der AdVance™-Schlinge (Boston Scientific, Marlborough, MA, USA) notwendig ist um gute Ergebnisse zu erzielen. Diese Zentrumsbildung hat zwei Vorteile. Zum einen erhöht die Expertise das Bewusstsein in der Region, inkontinente Männer eher einer Therapie im entsprechenden Zentrum zuzuführen. Zum anderen führt die Zentrumsbildung auch zu einem hohen Anteil überregionaler Patienten.

Limitation dieser Arbeit ist die nicht absolut exakte Einordnung der OPS-Kodierung. Wie in der Methodik beschrieben existieren mehrere Kodes für Schlingenimplantationen. Es können unterschiedliche Produkte nicht voneinander abgegrenzt werden. Weiterhin ist in der Methodik die Abgrenzung zwischen Schlingenimplantationen zwischen Mann und Frau dargestellt. Eine weitere Limitation ist die Abhängigkeit der Kodierung von der Fachabteilung. Insbesondere adjustierbare Schlingensysteme wurden teilweise als artifizielle Sphinkter verschlüsselt. Diese Entwicklung war in den letzten Jahren wieder rückläufig und hat keinen Einfluss auf diese Gesamtdarstellung. Weiterhin ist einschränkend zu betrachten, dass jährliche Fallzahlen von 1–3 Eingriffen als 1 anonymisiert und zusammengefasst werden. Dementsprechend ist eine exakte Differenzierung der niedrigen Fallzahlen nicht möglich. Zudem können Patienten, die bereits eine Inkontinenztherapie erhalten hatten, nicht sicher identifiziert werden. Auch die Gründe für den Rückgang der Schlingenimplantationen können mit diesen Daten nicht exakt dargestellt werden und es verbleiben die obigen zu diskutierenden Punkte. Nichtsdestotrotz ermöglicht die Auswertung von Qualitätsberichtsdaten der Krankenhäuser ein breites Bild der realen Versorgungssituation in Deutschland.

Diese detaillierte Analyse der strukturellen Versorgungssituation von harninkontinenten Männern zeigt erstmalig den deutlichen Rückgang von Schlingenoperationen in Deutschland auf. Dieser Rückgang ist insbesondere in kleinen Kliniken zu verzeichnen und wurde nicht durch den Anstieg anderer Inkontinenzoperationen kompensiert. Somit ist zu vermuten, dass es einen relevanten Anteil an nicht therapierten inkontinenten Männern gibt, die vor 10 Jahren noch für Schlingenoperationen gewonnen werden konnten.

## Fazit für die Praxis

- Die Implantation von Schlingen und Schlingensystemen hat in den letzten 10 Jahren deutlich abgenommen.
- Mögliche Gründe sind neben einer differenzierteren Indikationsstellung auch negative Erfahrungen nach anfänglichem „sling hype“ nach Einführung der verschiedenen Schlingensysteme.
- Der Rückgang von Schlingenoperationen ist insbesondere in kleinen urologischen Kliniken zu verzeichnen.
- Aufgrund des deutlichen Rückgangs an Inkontinenzoperationen beim Mann ist ein Versorgungsdefizit zu vermuten.
